# Feasibility of an adjustable-angle arthroscope during ACL femoral tunnel creation: an *ex vivo* porcine knee study

**DOI:** 10.3389/fsurg.2026.1899167

**Published:** 2026-09-10

**Authors:** Zhenghui Hu, Feng Zhou, Peng Su, Ruizhe Bai, Jian Li

**Affiliations:** 1 Department of Orthopaedic Surgery, The Second Affiliated Hospital of Soochow University, Suzhou, Jiangsu, China; 2 Department of Orthopaedic Surgery, Shanghai Seventh People's Hospital, Pudong New Area, Shanghai, China

**Keywords:** adjustable-angle arthroscope, anterior cruciate ligament, CT three-dimensional reconstruction, *ex vivo* porcine knee model, feasibility, femoral tunnel position

## Abstract

**Objective:**

Building on prior device development and bench-based visual-field validation, this exploratory study evaluated the feasibility of an adjustable-angle arthroscope for ACL femoral tunnel creation in an *ex vivo* porcine knee model and descriptively compared femoral tunnel position measurements and procedural time with a conventional 30° arthroscope.

**Methods:**

Ten fresh porcine knee specimens were allocated to an adjustable-angle arthroscope group or a conventional 30° arthroscope group (*n* = 5 each). The operational target point was defined as the intersection 5 mm from the inferior and posterior cartilage margins of the medial wall of the lateral femoral condyle; this standardized experimental target was not assumed to represent the native porcine or human ACL footprint center. All procedures were performed by one orthopaedic surgeon who was experienced in ACL reconstruction and was a member of the device-development team. CT-based three-dimensional reconstruction and gross anatomical measurement were used to determine distances A and B, and operative time and measurement reliability were recorded. Group-level outcomes were summarized descriptively.

**Results:**

On CT three-dimensional reconstruction, distances A and B were 4.93 ± 0.79 mm and 7.27 ± 0.85 mm, respectively, in the adjustable-angle group and 6.68 ± 1.15 mm and 8.98 ± 1.17 mm, respectively, in the 30° group. Gross anatomical measurements were 4.9 ± 0.7 mm and 7.1 ± 0.8 mm in the adjustable-angle group and 6.7 ± 1.2 mm and 9.0 ± 1.3 mm in the 30° group. Mean operative time was 14.23 ± 1.00 min and 14.46 ± 1.21 min, respectively. Interobserver ICCs were 0.91 and 0.90, and intraobserver ICCs were 0.97 and 0.97.

**Conclusion:**

This study successfully developed an adjustable-angle arthroscope based on a hinge-linkage mechanism and demonstrated, through quantitative testing, that it provided greater visual-field coverage than conventional 30° and 70° fixed-angle arthroscopes. By enabling dynamic multiangle visualization within a single arthroscopic system, this device may provide a more flexible visualization strategy for complex arthroscopic procedures and has potential clinical applicability.

## Introduction

Arthroscopy has become a central diagnostic and therapeutic modality in orthopaedic surgery and sports medicine because it combines minimal invasiveness with high-definition visualization and dynamic assessment of intra-articular structures. ACL injury is one of the most common and functionally consequential knee injuries in sports medicine, and treatment aims not only to restore anterior stability but also to recover rotational stability and reduce secondary meniscal and chondral injury (1). The increasing rate of ACL reconstruction further underscores the clinical burden of this procedure in contemporary sports medicine (2). Accordingly, refinements in the key technical steps of ACL reconstruction remain important for improving arthroscopic surgical quality.

The outcome of ACL reconstruction depends heavily on accurate recognition of relevant anatomy and precise tunnel placement. The ACL is a complex functional structure rather than a simple isometric band, and the femoral insertion site directly influences graft biomechanics and knee kinematics (3). The core principle of anatomic ACL reconstruction is restoration of the native footprint and its functional relationship, rather than merely achieving graft fixation (4). The quadrant method provides a classic radiographic framework for quantifying the femoral insertion site and has helped shift ACL reconstruction from experience-based tunnel placement toward anatomy-based positioning (5).

Malposition of the femoral tunnel is widely recognized as an important technical cause of ACL reconstruction failure and revision surgery. Femoral tunnel malposition has been identified as one of the most common technical factors associated with failure of primary ACL reconstruction in the MARS multicenter revision cohort (6). Technical errors, particularly nonanatomic tunnel placement, are also major contributors to residual instability and revision surgery (7). From a clinical kinematic perspective, vertical femoral tunnel placement can increase postoperative rotational laxity, indicating that even subtle deviations in femoral tunnel position may translate into clinically relevant functional consequences (8). Therefore, reliable intraoperative visualization of the femoral footprint and surrounding osseous and cartilaginous landmarks is essential for accurate femoral tunnel creation.

At present, the 30° arthroscope remains the most commonly used viewing instrument in ACL reconstruction; however, its fixed viewing angle may limit visualization of the femoral footprint and adjacent anatomic landmarks under certain portal and working conditions. Transtibial drilling has inherent anatomic limitations for anatomic femoral tunnel creation, often requiring independent femoral tunnel techniques, altered viewing portals, or different arthroscopic viewing angles to improve tunnel localization (9). To expand visualization, 70° arthroscopes have been used for viewing the femoral footprint during ACL reconstruction, and a 70° arthroscope combined with a flexible reamer system may improve visualization of native ACL anatomy and support more independent creation of femoral and tibial tunnels (10). Nevertheless, fixed high-angle arthroscopes may increase the spatial-orientation burden on surgeons and cannot provide continuous, dynamic visual adjustment throughout different stages of the procedure.

To address the limited field of view and restricted intraoperative flexibility of conventional fixed-angle arthroscopes, our group previously designed and fabricated an adjustable-angle arthroscope with a distal viewing angle continuously adjustable from 0° to 90°. The device changes the viewing direction through a distal angle-control mechanism, aiming to preserve the intuitive forward orientation of low-angle arthroscopes while providing the ability of high-angle arthroscopes to visualize hidden areas. Preliminary standardized bench testing demonstrated that the adjustable-angle arthroscope provided broader field-of-view coverage than conventional 30° and 70° fixed-angle arthroscopes, suggesting that it may offer a more flexible and continuous visualization strategy for complex arthroscopic procedures. Therefore, the present exploratory study used fresh porcine knee specimens to establish an ACL femoral tunnel model and descriptively compared femoral tunnel position measurements and operative time between an adjustable-angle arthroscope and a conventional 30° arthroscope using CT three-dimensional reconstruction and gross anatomical measurement.

## Materials and methods

### Study design

Fresh porcine knee specimens were used to create an ACL femoral tunnel model. Specimens were allocated to an adjustable-angle arthroscope group or a conventional 30° arthroscope group. Both groups underwent femoral tunnel creation according to an identical surgical workflow, and operative time was recorded. Postoperative CT three-dimensional reconstruction and gross anatomical measurement were used to evaluate distances A and B from the tunnel center to the inferior and posterior cartilage margins. Outcomes comprised the group-level CT and gross anatomical distances, operative time, and measurement reliability. The 5-mm target was an operational experimental reference and was not assumed to represent the native porcine or human ACL footprint center. The placement outcomes were interpreted descriptively rather than as a formal test of superiority. The retained study records did not describe sequence generation, allocation concealment, or procedural order; allocation is therefore reported without claiming randomization.

### Arthroscopic instruments

**Figure 1:**
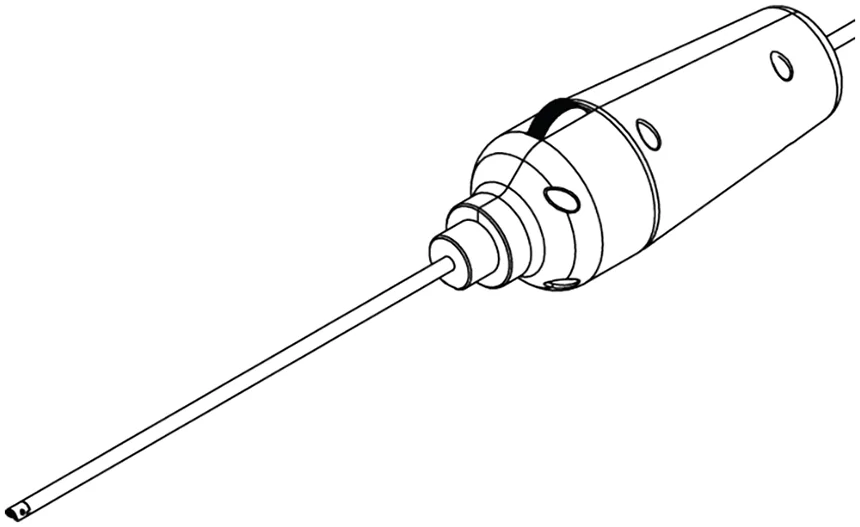
Design schematic of the adjustable-angle arthroscope. The distal viewing angle can be continuously adjusted through a handle-based control mechanism.

The adjustable-angle arthroscope used in this study was a prototype device previously designed and fabricated by our research team (Figure 1). The system consisted of the arthroscope body, an adjustable distal viewing module, a handle-based control unit, an illumination component, and an image-acquisition system. According to the design specifications, the outer diameter was 4.0 mm; the distal viewing angle was continuously adjustable from 0° to 90°, and the field of view was 120° ± 6°. Distal angulation was controlled through a handle-based gear mechanism and hinged linkage structure, allowing the surgeon to change the viewing direction in real time. The distal linkage deflected within a single principal plane; additional directional viewing required rotation of the arthroscope shaft. Shaft rotation caused corresponding rotation of the displayed image. The prototype did not include automatic image leveling, stabilization, or inversion correction, so image orientation had to be re-established by the operator after shaft rotation or major angle adjustment. The imaging end incorporated a miniature camera module and LED illumination component connected to a unified image-management and display system.

To minimize the influence of differences in imaging conditions, both groups underwent femoral tunnel creation using the same light source, display system, image-recording system, white-balance settings, and gain settings. Except for the method of viewing-angle adjustment, the instrument environment, specimen fixation, surgical portals, and femoral tunnel creation workflow were kept as consistent as possible between groups.

### Specimen acquisition and grouping

Ten fresh adult porcine knee specimens were included. All specimens were obtained from healthy domestic pigs aged at least 7 months and weighing no less than 90 kg. The limbs were transected at the hip and ankle levels, preserving the intact knee joint and surrounding soft tissues. Specimens were inspected before inclusion, and knees with visible anatomic deformity, osseous injury, ligament injury, cartilage defect, or other joint abnormality were excluded.

All specimens were maintained in a fresh state before testing and allowed to reach room temperature before surgery. The 10 knees were allocated to two groups of five specimens each. In the adjustable-angle arthroscope group, femoral tunnels were created with the prototype 0°–90° adjustable-angle arthroscope. In the conventional group, femoral tunnels were created with a standard 30° fixed-angle arthroscope.

### Surgical procedure and femoral tunnel creation

To reduce procedural confounding, the fixation method, portals, target-point definition, femoral guide, and drilling workflow were identical in both groups. All procedures were performed by the same orthopaedic surgeon, who was experienced in ACL reconstruction, participated in development of the adjustable-angle arthroscope, and was familiar with the prototype before the formal experiment. Prior to the formal test, participant completed three practice trials using the adjustable-angle arthroscope.

**Figure 2:**
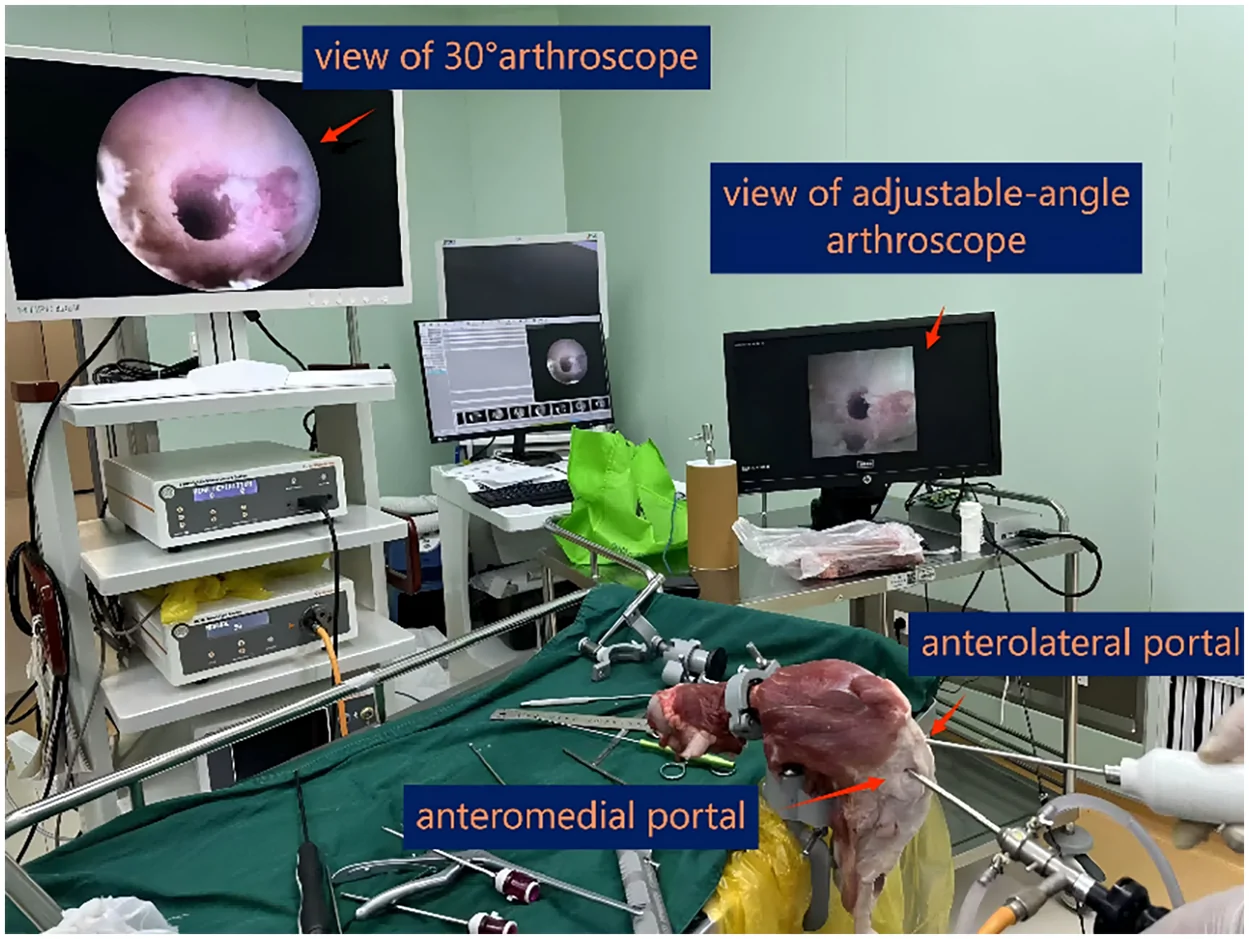
Porcine knee fixation and arthroscopic operating platform. The proximal femur was secured in a frame that allowed 0°–120° of knee flexion, and femoral tunnels were created through standard portals in both groups.

Before surgery, each porcine knee was secured in a stable operating frame, with the proximal femur fixed and the knee allowed to move through 0° to 120° of flexion to simulate the joint manipulation commonly used during ACL reconstruction (Figure 2). The same fixation method and surgical position were used for all specimens to minimize the effect of specimen positioning on tunnel localization. Standard anteromedial and anterolateral portals were then established. After entry into the joint, the ACL was excised, and radiofrequency ablation and shaver debridement were used to clear soft tissue around the femoral insertion site, fully exposing the medial wall of the lateral femoral condyle, the intercondylar notch, and the surrounding cartilage margins.

The femoral tunnel target point was defined operationally with reference to the cartilage margins surrounding the femoral ACL footprint. The target tunnel center was set at the intersection 5 mm from the inferior cartilage margin and 5 mm from the posterior cartilage margin of the medial wall of the lateral femoral condyle. This standardized experimental point was selected because it was practical within the porcine intercondylar notch and approximated the length of the curved tip of a standard arthroscopic probe, thereby reducing variation from unaided visual estimation. The inferior and posterior cartilage margins served as reproducible landmarks after ACL excision. In the conventional group, the surgeon used a 30° fixed-angle arthroscope to visualize these cartilage margins and the predefined target point. In the adjustable-angle group, the surgeon adjusted the distal viewing angle in real time to visualize the same landmarks and target-point region. An inside-out anteromedial-portal drilling technique was used in both groups. The same femoral guide and drilling method were used to minimize between-group procedural confounding.

After femoral tunnel creation, operative time was recorded from completion of arthroscopic portal establishment to completion of femoral tunnel drilling. The occurrence of posterior wall blowout, tunnel discontinuity, cartilage damage, or other procedure-related complications was also recorded.

### CT three-dimensional reconstruction and measurement

**Figure 3:**
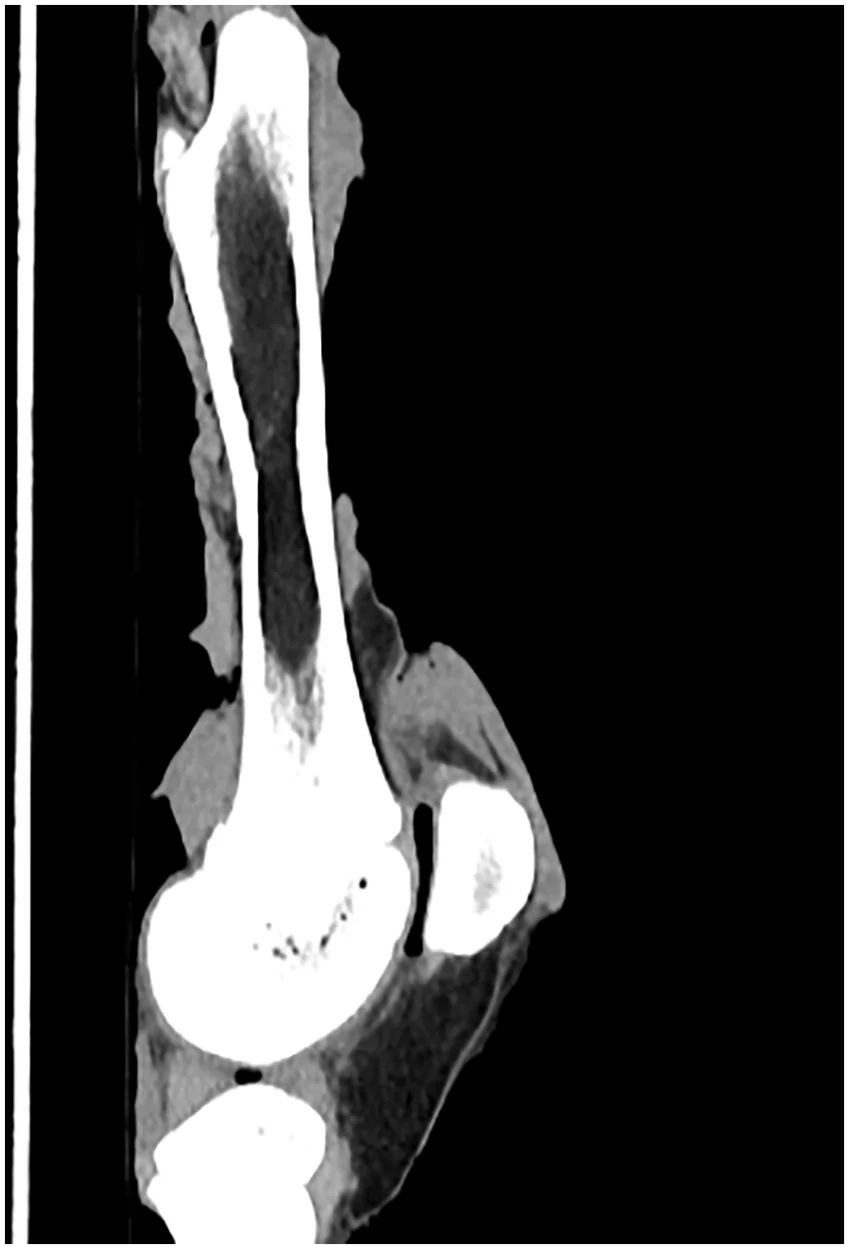
Standardized lateral-view correction before CT three-dimensional reconstruction. The posterior contours of the medial and lateral femoral condyles were overlapped to obtain a consistent measurement plane.

After femoral tunnel creation, all porcine knee specimens underwent CT scanning to evaluate femoral tunnel position. The image data were imported into 3D Slicer software (version 5.6.2) for three-dimensional reconstruction. During reconstruction, the model was first adjusted in transparent display mode to a standardized lateral view by overlapping the posterior contours of the medial and lateral femoral condyles, thereby obtaining a consistent measurement plane (Figure 3). The distal femur was then reconstructed, and the soft tissues, patella, tibia, and fibula were progressively removed by segmentation and cropping to fully expose the intercondylar notch, medial wall of the lateral femoral condyle, and femoral tunnel aperture.

**Figure 4:**
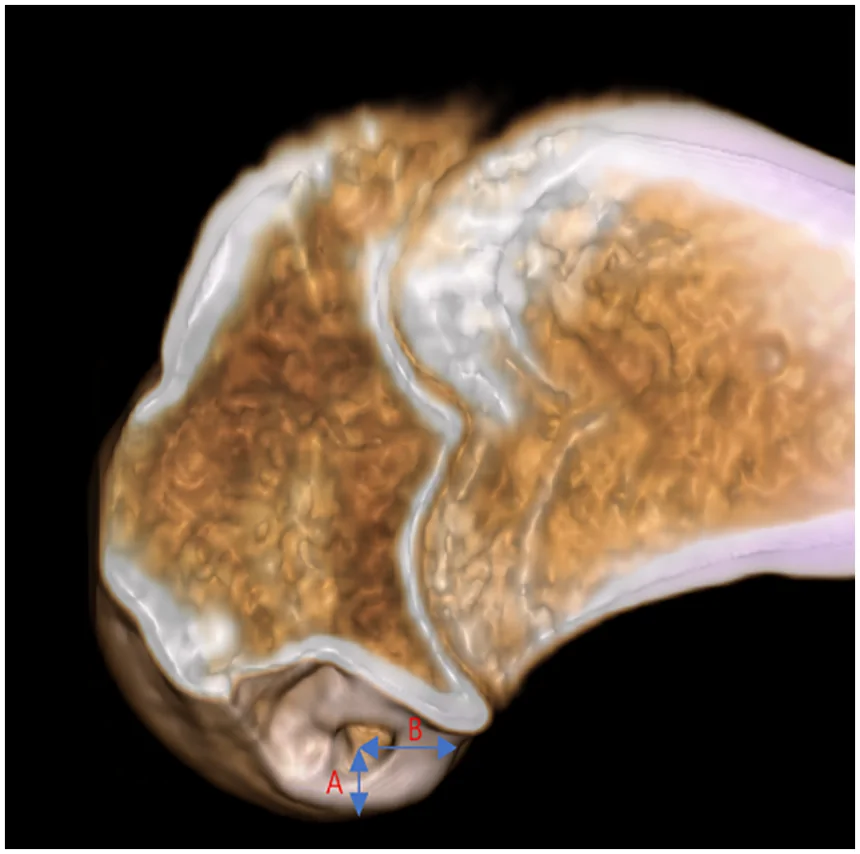
CT three-dimensional reconstruction of the distal porcine femur and femoral tunnel location, used to measure distances A and B between the tunnel center and the inferior and posterior cartilage margins.

The three-dimensional model was further adjusted until the intercondylar roof was aligned with the Blumensaat line. The inferior and posterior cartilage margins were identified as the visible boundaries of the articular cartilage on the standardized medial wall of the lateral femoral condyle, and the tunnel center was defined as the geometric center of the tunnel aperture. The distance from the tunnel center to the inferior cartilage margin and the distance from the tunnel center to the posterior cartilage margin were measured (Figure 4). All reconstructions and measurements were performed according to the same workflow to ensure consistency of the measurement plane and landmark definitions across specimens.

For subsequent analysis, the distance from the femoral tunnel center to the inferior cartilage margin of the medial wall of the lateral femoral condyle was defined as distance A, and the distance from the femoral tunnel center to the posterior cartilage margin was defined as distance B. These distances were summarized at group level and interpreted descriptively relative to the operational 5-mm reference. All measurements were performed under standardized three-dimensional reconstruction and measurement conditions.

### Gross anatomical measurement

**Figure 5:**
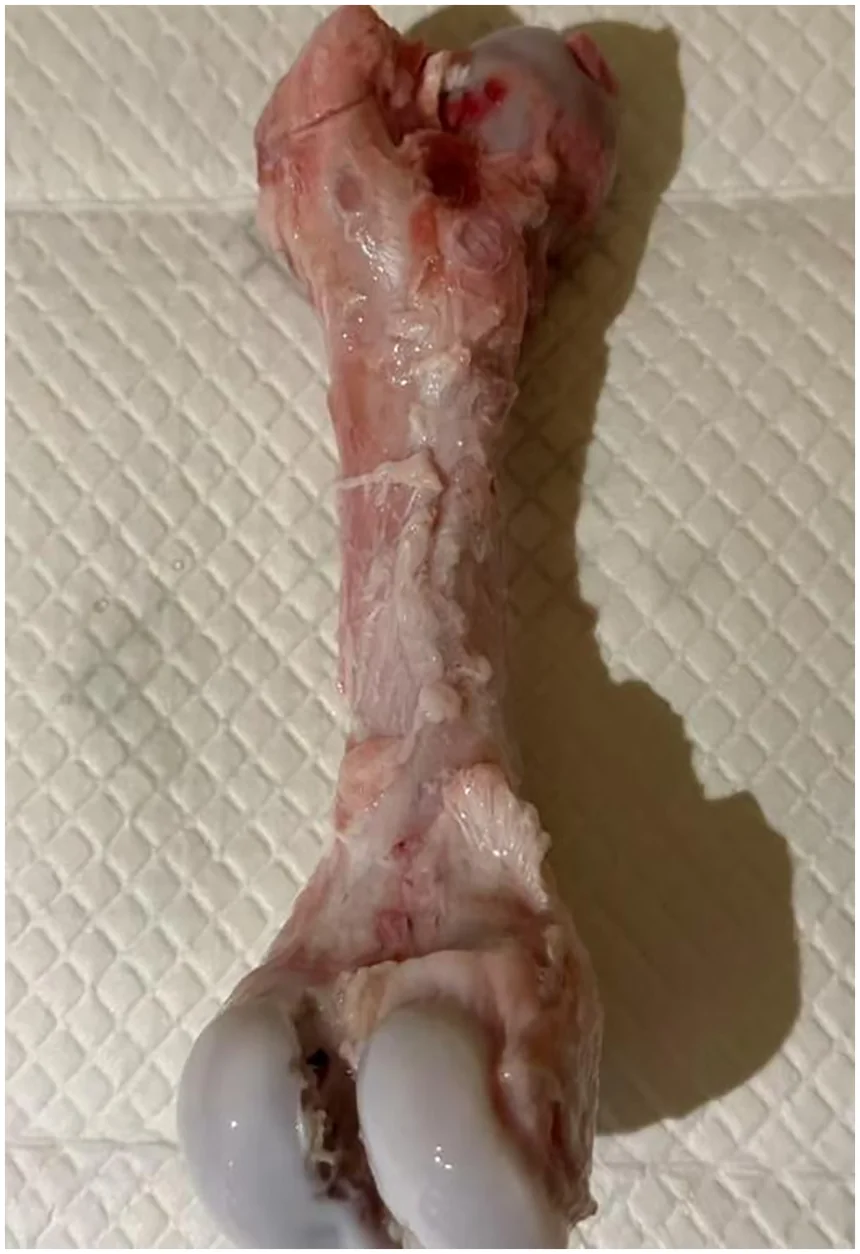
Gross anatomical exposure of the distal porcine femur, showing the intercondylar notch and femoral tunnel aperture.

After CT scanning and three-dimensional reconstruction, gross anatomical measurements were performed on all porcine knee specimens to further validate the imaging measurements. During dissection, the periarticular soft tissues, tibia, and other structures that interfered with observation were removed to fully expose the distal femoral intercondylar notch, the medial wall of the lateral femoral condyle, and the established femoral tunnel aperture (Figure 5). Care was taken to avoid damaging the tunnel aperture and surrounding cartilage margins.

**Figure 6:**
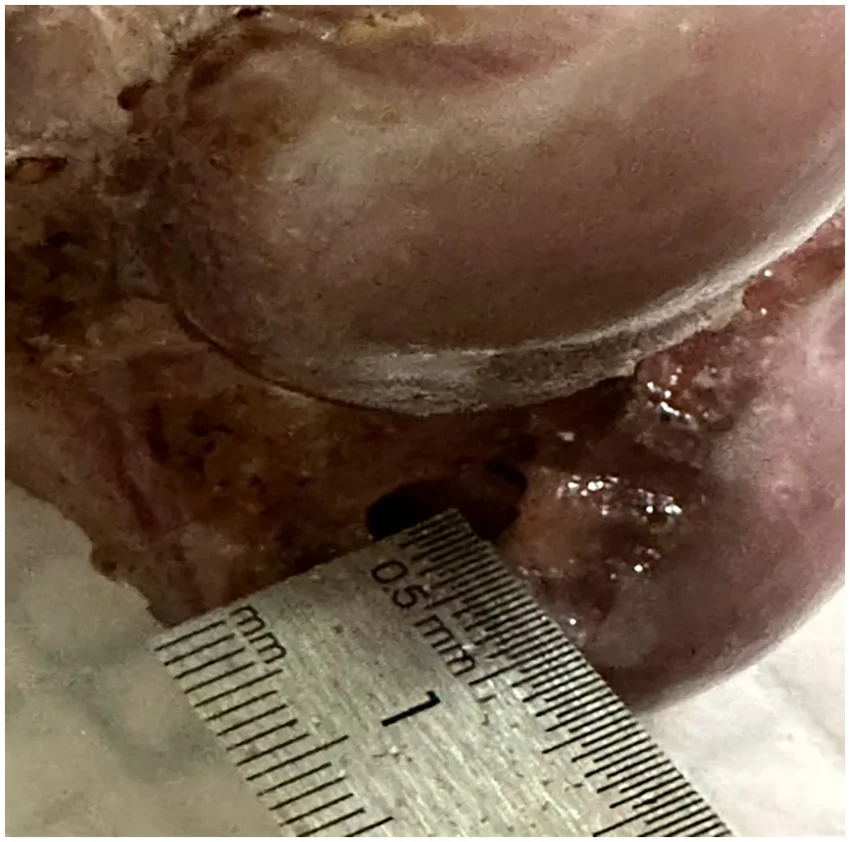
Gross anatomical measurement of the distance from the femoral tunnel center to the cartilage margin of the medial wall of the lateral femoral condyle using a ruler with 1-mm graduations.

After complete exposure of the tunnel aperture, the inferior and posterior cartilage margins of the medial wall of the lateral femoral condyle were used as reference landmarks, and the distances from the femoral tunnel center to these two margins were directly measured with a stainless-steel ruler with 1-mm graduations (Figure 6). Consistent with CT three-dimensional reconstruction, the distance from the tunnel center to the inferior cartilage margin was defined as distance A, and the distance from the tunnel center to the posterior cartilage margin was defined as distance B. Group-level gross anatomical results were reported to one decimal place to avoid implying precision beyond the measurement instrument.

All gross anatomical measurements were performed using the same measurement criteria and were compared with CT three-dimensional reconstruction measurements to evaluate agreement between the two methods. Gross anatomical measurements also served as an important reference for assessing the actual femoral tunnel position.

### Measurement reliability assessment

To assess measurement reliability, interobserver and intraobserver analyses were performed for CT three-dimensional reconstruction and gross anatomical measurements. All measurements were independently performed by three observers according to standardized criteria. Before measurement, all observers received identical training and were instructed on the definitions of distances A and B. Interobserver reliability was assessed by comparing measurements obtained by the three observers for the same specimens. To assess intraobserver reliability, one observer repeated all measurements 1 month after the initial measurement without access to the first set of results. ICCs with 95% confidence intervals were calculated. According to previously recommended criteria, ICC < 0.20 indicates poor agreement, 0.20 ≤ ICC < 0.40 indicates fair agreement, 0.40 ≤ ICC < 0.80 indicates strong agreement, and ICC ≥ 0.80 indicates excellent agreement.

### Statistical analysis

All statistical analyses were performed using the SPSSAU online statistical platform. Continuous variables are presented as mean ± standard deviation. Given the exploratory design and the small group size (*n* = 5 each), the CT, gross anatomical, and operative-time measurements are presented descriptively without formal between-group superiority or equivalence testing. Interobserver and intraobserver measurement reliability and CT–gross measurement agreement are reported as ICCs with the 95% confidence intervals available.

## Results

### Feasibility of adjustable-angle arthroscope–assisted femoral tunnel creation

A total of 10 fresh porcine knee specimens were included, with five specimens assigned to the adjustable-angle arthroscope group and five to the conventional 30° arthroscope group. Arthroscopic portal establishment, ACL excision, exposure of the femoral footprint, and femoral tunnel drilling were completed successfully in all specimens. No procedure was discontinued because of instrument interference, loss of visualization, or portal-related limitations.

All femoral tunnels were created successfully and remained continuous and intact. No posterior wall blowout, tunnel discontinuity, severe cartilage injury, or other major drilling-related complication was observed.

### CT three-dimensional reconstruction measurements of femoral tunnel position

Postoperative CT three-dimensional reconstruction demonstrated the femoral tunnel aperture and its spatial relationship to the inferior and posterior cartilage margins of the medial wall of the lateral femoral condyle in both groups. Group-level distances are reported descriptively below.

**Figure 7:**
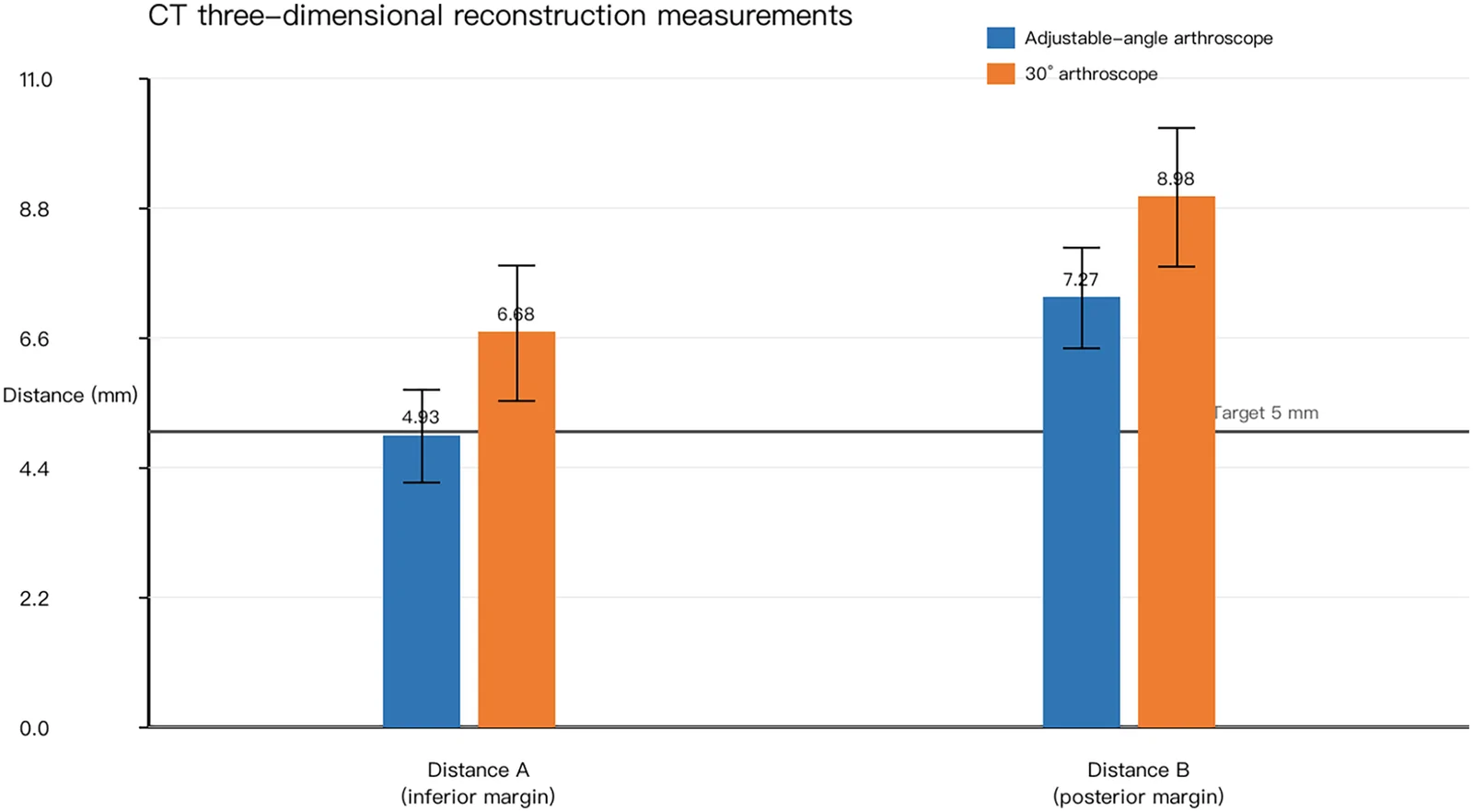
CT three-dimensional reconstruction measurements of distances A and B. The horizontal line indicates the operational 5-mm reference. Group means are shown descriptively; no specimen-level placement-error comparison was performed.

In the adjustable-angle arthroscope group, distance A was 4.93 ± 0.79 mm and distance B was 7.27 ± 0.85 mm. In the conventional 30° arthroscope group, the corresponding distances were 6.68 ± 1.15 mm and 8.98 ± 1.17 mm (Figure 7).

### Gross anatomical measurements of femoral tunnel position

The femoral tunnel aperture was clearly identifiable in both groups. Tunnel morphology was intact, and no posterior wall blowout or abnormal tunnel exit was observed.

In the adjustable-angle arthroscope group, gross anatomical distance A was 4.9 ± 0.7 mm and distance B was 7.1 ± 0.8 mm. In the conventional 30° arthroscope group, the corresponding distances were 6.7 ± 1.2 mm and 9.0 ± 1.3 mm.

### Agreement between CT three-dimensional reconstruction and gross anatomical measurement

CT three-dimensional reconstruction measurements were compared with gross anatomical measurements. In the adjustable-angle arthroscope group, mean CT and gross measurements were 4.93 mm and 4.9 mm, respectively, for distance A and 7.27 mm and 7.1 mm, respectively, for distance B. In the conventional 30° arthroscope group, the corresponding values were 6.68 mm and 6.7 mm for distance A and 8.98 mm and 9.0 mm for distance B.

**Figure 8:**
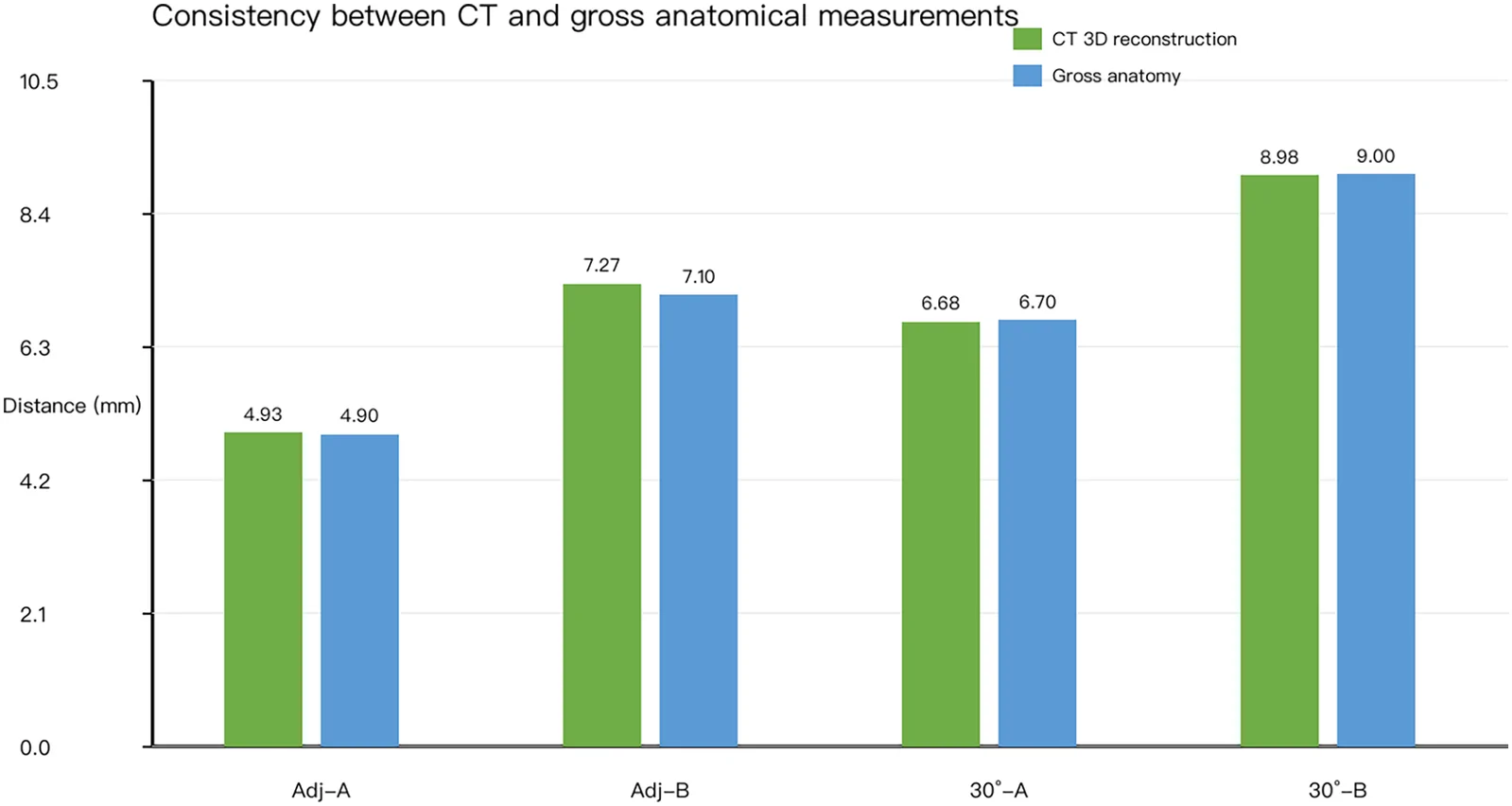
Comparison between CT three-dimensional reconstruction and gross anatomical measurements. Mean measurements and ICCs from the original analysis are shown; the ICCs do not exclude systematic measurement bias.

Agreement analysis demonstrated an ICC of 0.99 (95% CI, 0.98–0.99) between CT and gross anatomical measurements for distance A and an ICC of 0.99 (95% CI, 0.94–0.99) for distance B (Figure 8).

### Comparison of operative time

**Figure 9:**
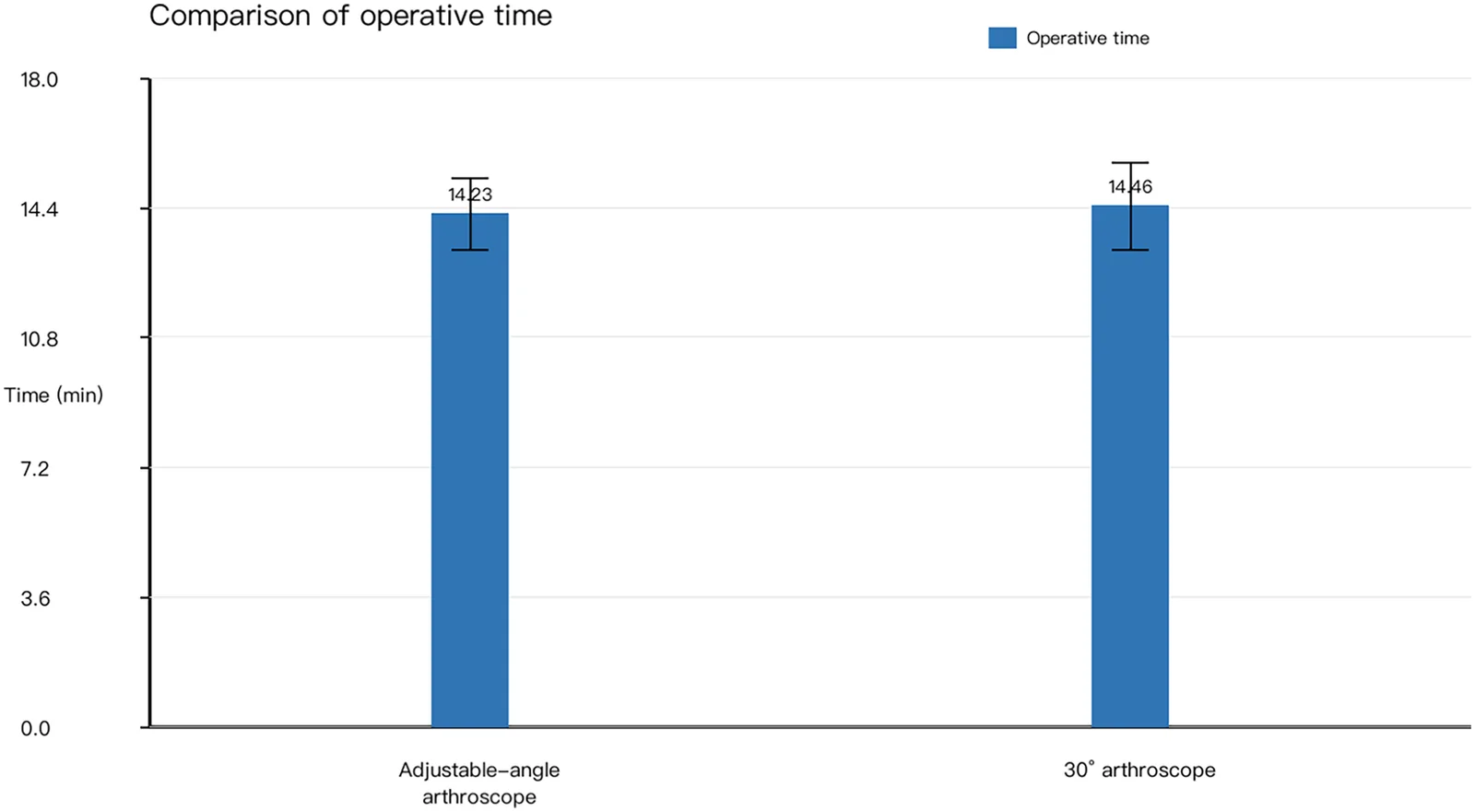
Descriptive comparison of mean operative time between groups. The displayed group means do not establish a difference or equivalence.

Mean operative time was 14.23 ± 1.00 min in the adjustable-angle arthroscope group and 14.46 ± 1.21 min in the conventional 30° arthroscope group (Figure 9).

### Measurement reliability

ICCs were used to evaluate interobserver and intraobserver reliability of femoral tunnel position measurements. Interobserver reliability analysis showed ICCs of 0.91 (95% CI, 0.77–0.98) for distance A and 0.90 (95% CI, 0.74–0.97) for distance B. Intraobserver reliability analysis showed ICCs of 0.97 for both distance A (95% CI, 0.87–0.99) and distance B (95% CI, 0.88–0.99). These results indicate high measurement reliability.

## Discussion

This exploratory study evaluated a prototype adjustable-angle arthroscope in a fresh porcine ACL femoral tunnel model after prior device development and bench-based visual-field validation. At group level, the mean CT and gross anatomical distances in the adjustable-angle group were numerically closer to the operational 5  ×  5-mm reference than those in the conventional 30° group. However, these summaries are descriptive and do not represent specimen-level absolute placement errors. Mean operative times were also descriptively similar. The findings support preliminary feasibility and justify further validation rather than establishing clinical effectiveness.

Within the conventional arthroscopic system, the 30° arthroscope is the most frequently used viewing instrument for ACL reconstruction but has inherent limitations for visualizing the femoral footprint. During femoral tunnel creation through the anteromedial portal, the arthroscope and femoral guide may compete for limited anterior working space, compromising viewing stability; when the arthroscope is placed through the anterolateral portal, the fixed 30° view may display the target region obliquely and impair spatial judgment (11). The 70° arthroscope has therefore been introduced to improve visualization of the femoral footprint, and an anteromedial portal technique combined with a 70° arthroscope can improve exposure of the medial wall of the lateral femoral condyle and the femoral footprint (12). However, although the 70° arthroscope expands the oblique field of view, the large divergence between the viewing direction and the shaft axis requires the surgeon to re-establish spatial orientation and may create new blind spots (13). Thus, a fixed-angle viewing system may not fully meet the need for dynamic visualization throughout the procedure.

The adjustable-angle arthroscope may help address these limitations by allowing continuous distal adjustment of the viewing direction. The inferior and posterior cartilage margins of the medial wall of the lateral femoral condyle served as reference landmarks in this experiment. Compared with fixed 30° and 70° arthroscopes, the adjustable-angle arthroscope permits real-time adjustment of the viewing angle while maintaining the same portal. A single fixed viewing angle may not accommodate the visualization requirements of all surgical stages (12, 13). By allowing smooth transition between low and high viewing angles, the device theoretically integrates aspects of 30° and 70° viewing.

The distal mechanism of the adjustable-angle arthroscope changes the viewing direction within a single bending plane, whereas multidirectional inspection requires rotation of the shaft. Because shaft rotation causes corresponding rotation of the displayed image and the prototype has no automatic leveling, stabilization, or inversion correction, superior–inferior and anterior–posterior screen orientation can shift during manipulation. The surgeon must therefore pause and re-establish orientation using visible anatomy and the known portal and instrument geometry after major shaft rotation or angle adjustment. This requirement may increase cognitive and visuospatial burden, particularly for inexperienced users.

Femoral tunnel position was evaluated by both CT three-dimensional reconstruction and gross anatomical measurement. CT three-dimensional reconstruction depicts the spatial relationship between the tunnel center and surrounding landmarks (14), and the two measurement methods showed ICCs of 0.99 in this study. The porcine knee also provides a practical experimental environment for arthroscopic manipulation and the evaluation of arthroscopic skills (15). A single-surgeon standardized workflow reduced interoperator variation, but this feature must be balanced against an important risk of bias: the surgeon participated in device development, could not be blinded to the arthroscope, and may have had greater familiarity or preference for the prototype. This design limits external validity and prevents separation of device performance from operator-specific effects.

The present findings provide preliminary experimental support for further evaluation of adjustable-angle arthroscopy during ACL femoral tunnel creation. Future studies should include larger cohorts, human cadaveric knees, prospective randomization of device order, blinded outcome assessment, and multiple independent surgeons to separate device effects from operator familiarity and quantify the learning curve. Preclinical safety, long-term mechanical stability, and imaging performance under irrigated surgical conditions should also be evaluated before clinical translation. Potential applications beyond ACL surgery remain hypotheses and require procedure-specific testing.

### Limitations

This study has several limitations. First, it used an *ex vivo* porcine knee model. Porcine joint dimensions, intercondylar notch morphology, cartilage thickness, and ligament insertion anatomy differ from those of human knees; therefore, the findings cannot be directly extrapolated to clinical ACL reconstruction. Second, the 5  ×  5-mm point was an operational experimental target selected for standardized testing and was not validated as the native porcine or human ACL footprint center. Third, the sample size was small (*n* = 5 per group), limiting precision and generalizability. Fourth, the reported group-level distances do not provide specimen-level absolute errors relative to 5 mm; the placement results are therefore descriptive and cannot establish superiority. Fifth, sex, body size, and individual anatomic variation were not controlled. Sixth, all procedures were performed by one unblinded surgeon who was part of the development team; this may introduce device-preference, performance, and learning-related bias despite reducing interoperator variation. Seventh, sequence generation, procedural order, assessor blinding, and pre-experiment practice count were not documented in the retained study records. Eighth, shaft rotation changed image orientation, no automatic correction was available, and neither cognitive burden nor the learning curve was quantified. Finally, the *ex vivo* model did not reproduce bleeding, variable irrigation, dynamic soft-tissue tension, patient-specific anatomy, or clinical safety outcomes.

Accordingly, the present results should be interpreted as preliminary experimental evidence of feasibility, not as proof of superior placement accuracy, clinical effectiveness, safety, equivalence, or ease of adoption. Larger animal studies, human cadaveric investigations, multi-surgeon validation, and preclinical safety testing are needed.

## Conclusion

On the basis of prior adjustable-angle arthroscope design and visual-field validation, this study further evaluated the device in a fresh porcine ACL femoral tunnel model. Compared with a conventional 30° arthroscope, the adjustable-angle arthroscope enabled femoral tunnel placement closer to the predefined target region, suggesting improved localization accuracy. The device did not meaningfully increase operative time, and CT three-dimensional reconstruction measurements showed excellent agreement with gross anatomical measurements. By allowing continuous adjustment of the viewing direction, the adjustable-angle arthroscope improved visualization of the medial wall of the lateral femoral condyle and the target region, providing a more flexible intraoperative visualization strategy for key steps requiring precise anatomic localization during ACL reconstruction. Further cadaveric and clinical studies are required to confirm its safety and clinical value.

## Data Availability

The raw data supporting the conclusions of this article will be made available by the authors, without undue reservation.
